# The Involvement of Human Factors Brings New Findings for Predicting Global Suitability Habitat for *Hyphantria cunea* (Lepidoptera: Arctiidae)

**DOI:** 10.1002/ece3.71421

**Published:** 2025-05-26

**Authors:** Haochang Hu, Hongwei Zhou, Yuxi Li, Yongzheng Li, Yunbo Yan, Jun Yang, Jun Chen, Yumo Chen, Di Cui

**Affiliations:** ^1^ College of Computer and Control Engineering Northeast Forestry University Harbin China; ^2^ Forestry Grassland Investigation and Planning Institute of Heilongjiang Province Harbin China; ^3^ Fengcheng Forestry Pest Control and Quarantine Station Fengcheng Forestry Development Service Center Fengcheng China; ^4^ School of Materials Science and Engineering Northeastern University Shenyang China; ^5^ Heilongjiang Forestry Technology Service Center Harbin China

**Keywords:** biological conservation, global suitability habitat, human factor, *Hyphantria cunea*, invasive pest

## Abstract

Invasive pests have spread globally at an unprecedented scale, severely threatening biodiversity and resulting in significant economic losses, emerging as a global problem. This study utilizes the Maxent model, incorporating human and natural factors to predict the current and future potential global distribution of *Hyphantria cunea* , for comparison with climate change. Results indicate that under the influence of climate change, human factors have significantly altered the potential global distribution of *H. cunea* . In contrast to the potential distribution driven by climate change, this paper suggests that the suitable habitat area for *H. cunea* in Oceania, Southern Hemisphere, is expected to increase. Over the long term, under the SSP126 and 585 scenarios, there is a forecasted reduction of 25.2% and 33.2% in the suitable living area for *H. cunea* , whereas the SSP245 and 370 scenarios anticipate increases of 13.9% and 5.7%, respectively. Moreover, this research identifies areas of high suitability across continents and forecasts changes in the distribution patterns of *H. cunea* in the future. It offers crucial insights for developing more effective global quarantine strategies and pest management policies.

## Graphical abstract

Distribution of *Hyphantria cunea* over the next 40 years and changes in highly suitable habitat across continents. (a) SSP126; (b) SSP245; (c) SSP370; (d) SSP585.

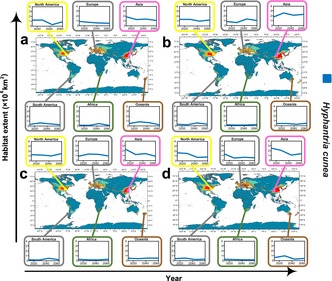

## 1 Introduction

**FIGURE 1:**
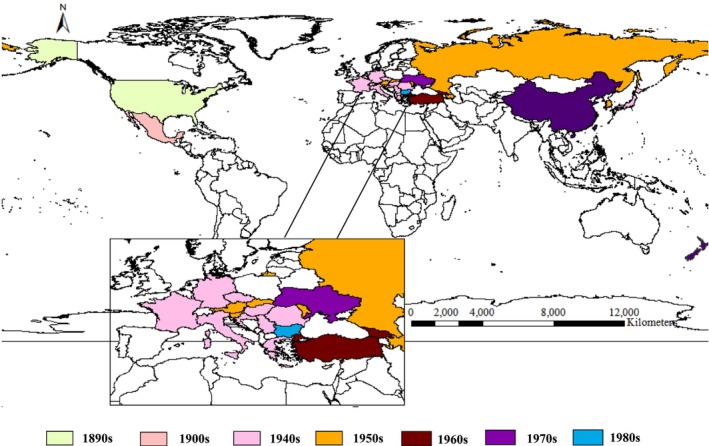
The global distribution of *Hyphantria cunea* across various countries and regions.

As human activities and climate changes are affecting species distributions and ecosystem stabilities, leading to the global spread of invasive pests at an unprecedented scale, which has become a worldwide issue (Hulme 2021; Wallingford et al. 2020). The Intergovernmental Science‐Policy Platform on Biodiversity and Ecosystem Services (IPBES) shockingly exposes that the introduction and expansion of species becoming invasive in new environments is a leading cause of the current collapse in biodiversity (Díaz et al. 2019). Invasive pests represent a significant threat to biodiversity and lead to considerable economic damages (Strubbe et al. 2023). Although it is crucial to limit the spread of invasive pests, prevention of their introduction continues to be the most effective method of control. Consequently, it is urgently necessary to find reliable methods for predicting invasions before they occur in order to adjust management policies and reduce the likelihood of invasions (Wylie and Janssen‐May 2017). This emphasizes the importance of determining potential suitable habitat for invasive pests beforehand. The *Hyphantria cunea* (Lepidoptera: Arctiidae) originating from North America is recognized globally as a quarantine pest and is known for its rapid dispersion (Sun et al. 2021; Gao et al. 2022; Xu et al. 2023). The first indications of *H. cunea* 's harmful presence were noted in the United States in 1899, spreading to Central Europe and East Asia in the 1940s and expanding to over 20 countries globally by 1972 (Gomi et al. 2009; Choi and Park 2012), as illustrated in Figure 1.

Feeding on more than 600 host plants including fruit trees, shrubs, herbs, and crops, the *Hyphantria cunea* inflicts substantial economic damage on agricultural and forestry production (Edosa et al. 2018). In Asia, where the infestation of the *H. cunea* is most severe globally, the losses caused in 2021 reached $1.605 billion, of which the economic losses were approximately $200 million, and the non‐economic losses were $1.405 billion. With a broad range of host plants and high fecundity, the *H. cunea* exhibits significant ecological adaptability, complicating management (Wang et al. 2021; Li et al. 2022). Consequently, preemptive risk assessment of the *H. cunea* propagation is essential to formulate effective pest control measures and plant protection strategies (Ye et al. 2024). Currently, numerous studies have been undertaken to predict the potential future geographic distribution of *Hyphantria cunea* . Research from New Zealand has constructed a seasonal climate cohort model for *H. cunea* to forecast its outbreaks and potential invasion trends (Kean and Kumarasinghe 2007). In two separate studies focused on China, the actual occurrences of *H. cunea* were examined, delineating its suitable habitats and patterns of ecological niche shifts, and unveiling its dispersal rules (Tang et al. 2021; Wen et al. 2024). Studies on human factors used nighttime light intensity to simulate human transmission capabilities, revealing the spread pattern of *H. cunea* in China (Ye et al. 2024). A global predictive study on how climate affects the potential distribution of *H. cunea* has been initiated (Ge et al. 2019).

There are two limitations to these studies mentioned above: (1) The research scope is confined to the researchers' own countries and regions, resulting in a localized analysis and a lack of extensive consideration of the global dynamics of *H. cunea* . (2) The focus of the research primarily lies on environmental factors, particularly assessing the suitability of *H. cunea* under climate change influences, neglecting the role of human factors in the analysis of global habitat suitability. Earlier research shows that *Hyphantria cunea* 's dispersal primarily relies on natural factors for short distances and human activities for long distances (Ye et al. 2024). Environmental changes caused by human activities expose organisms (including pests) to heightened sensory stimuli, leading to novel behavioral reactions (Du et al. 2018). Human factors play a crucial role in pest dispersal; thus, significant human activities should be included in global early warning systems to optimize risk assessments (Lippitt et al. 2008). Nighttime light intensity frequently serves as an indicator of human activity, mirroring economic circumstances (Delibes et al. 2001; Wu et al. 2023). Moths are drawn to light sources like streetlights, potentially altering their behaviors (Degen et al. 2016; Van Langevelde et al. 2017; Cieraad et al. 2022; Grubisic et al. 2018; Owens et al. 2020). Similarly, nighttime light intensity is considered a contributing factor directly influencing the distribution of moth communities (LaRoe et al. 2022). The interplay of human factors with climate change in predicting the distribution of *H. cunea* is understudied, resulting in few research efforts in this field. This results in a degree of bias in the studies predicting the distribution of *H. cunea* , constraining their practicality. Thus, assessing the risk of *H. cunea* must accurately account for the effects of human activities on its spread, beyond just climate suitability analysis (Ye et al. 2024).

Species Distribution Models (SDM) employ geographic reference points, variable digital maps, and related algorithms to estimate the potential distribution and spatial quantities of pests (Méndez‐Vázquez et al. 2019). Recent research commonly uses SDM for risk assessments of harmful species (Dessie et al. 2024), predicting the potential spread of these species beyond their current geographical ranges by combining their distributions with variable factors (Mainali et al. 2015). Assessing suitable environments and risk areas for invasive species has proven to be an effective method for predicting the potential geographic distribution of agricultural and forestry pests (Grünig et al. 2020; Ikeda and Osawa 2020). The concentration of atmospheric CO2 in Global Climate Models (GCM) and Shared Socioeconomic Pathways (SSP) influences the uncertainty and changes in predictive behaviors (Buisson et al. 2010; Moss et al. 2010). In the plethora of available SDMs, the Maxent model excels above others as it delivers greater accuracy with only limited data samples (Guo et al. 2024). In contrast, the CLIMEX model focuses more on simulating the responses of species to climatic variables, whereas in this study, we are more concerned with the impact of human factors on the potential distribution of the *Hyphantria cunea* . Consequently, it is widely used to estimate the potential distribution of global quarantine pests such a*s Bursaphelenchus xylophilus* (Ikegami and Jenkins 2018), *Lycorma delicatula* (Wakie et al. 2020), and *Solenopsis invicta Buren* (Li, Li, et al. 2023). Current research based on SDMs primarily focuses on predicting the suitability distribution of pests based on environmental factors. However, the spatial distribution of pests is not only constrained by natural conditions but also influenced by human factors. Although previous research has considered the dynamic changes in global suitability for *Hyphantria cunea* due to climate change (Ge et al. 2019), their findings are expected to differ significantly from those influenced by human factors. In order to better comprehend how changes in natural factors (such as temperature, precipitation, normalized vegetation index) and human factors (such as human footprint, nighttime light intensity) jointly affect the future global suitability habitat of *H. cunea* , the aims of this study are given as follows: (1) To evaluate the importance of impact indicators on the current global distribution of *H. cunea* influenced by both natural and human factors; (2) To predict shifts in the potential global distribution of *H. cunea* and analyze critical suitable habitat; (3) To reveal the impacts of human and natural factor changes on the global distribution patterns of *H. cunea* .

This research can guide quarantine strategies globally and provide a scientific basis and practical methods for the management and control of other pests and diseases. The results not only assist in current epidemic management but also strengthen future defenses against similar global invasive pests.

## 2 Materials and Methods

### 2.1 Collection of *Hyphantria cunea* Distribution Data

**FIGURE 2:**
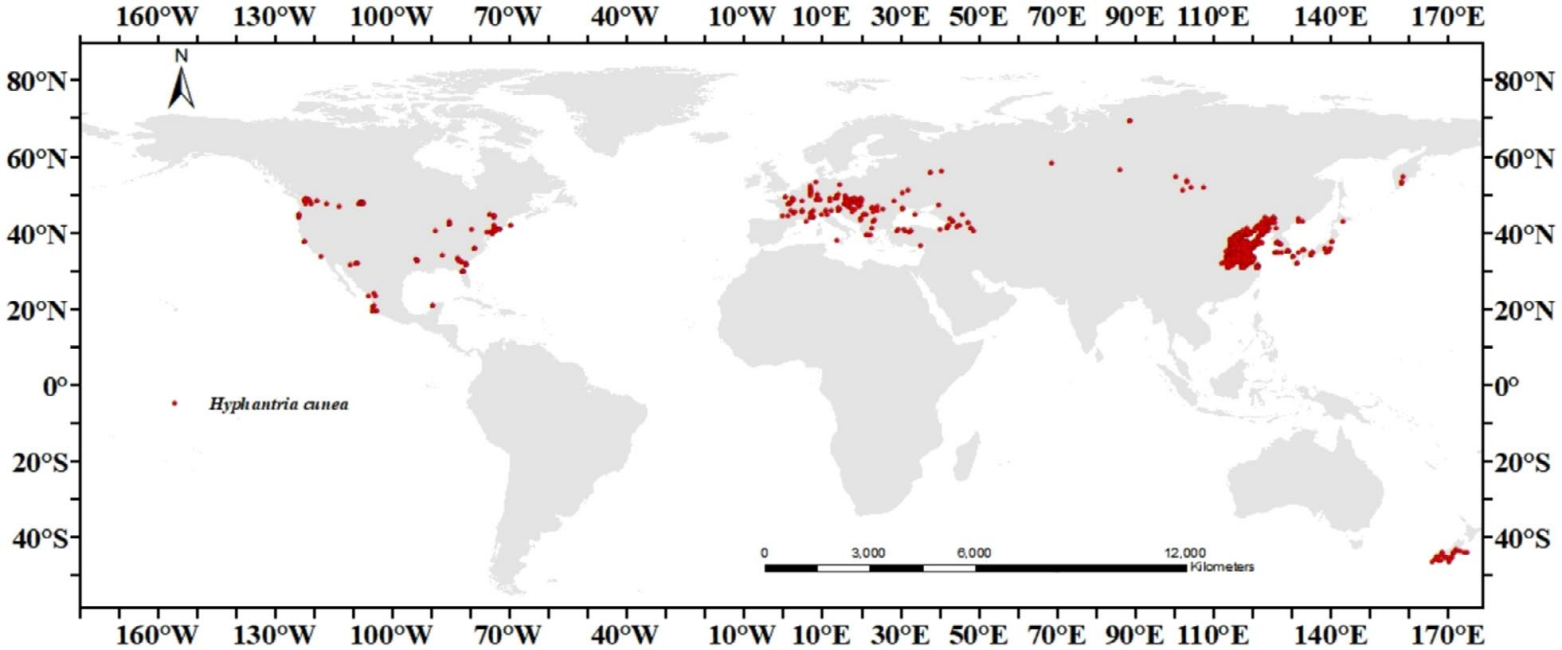
Global distribution data points of *Hyphantria cunea* .

This study collected data by accessing the Global Biodiversity Information Facility (GBIF, accessed March 2024), Barcode of Life Data Systems (BOLD, accessed March 2024), and the invasive species catalog of the Centre for Agriculture and Bioscience International (CABI‐ISC, accessed March 2024). To ensure the quality and consistency of the data, we first exported data from each source and then removed duplicate records, corrected obvious errors, and resolved inconsistencies in the entries. Next, we verified and standardized the species identification information for *H. cunea* , ensuring that the species nomenclature across all data sources was consistent with the International Species Information System. All geographic points that were not on the map were deleted to decrease spatial autocorrelation and sampling bias (Zhao et al. 2023). For data obtained from GBIF and BOLD, we verified the reasonableness of the collection dates and locations. Based on spatial debias sampling, we used the spThin and weight.env functions in the SDMTools package to perform spatial sparsity of the original occurrence points and weight the kernel density of the environmental variables, setting a minimum distance threshold of 5 km to ensure the spatial independence of the training samples and reduce the model bias in densely sampled areas. After such a filtering process, this study obtained a distribution dataset containing 923 unique geographic locations, as shown in Figure 2.

In summary, the point distribution data were obtained by accessing the Global Biodiversity Information Facility (GBIF, accessed in March 2024), the Barcode of Life Data System (BOLD, accessed in March 2024), and the CABI Invasive Species Compendium (CABI‐ISC, accessed in March 2024). For the determination of pseudo‐absence data, due to the difficulty in obtaining actual absence points, the “random” command in Biomod2 was used to generate 10 sets of 1000 pseudo absence points. The weights of the presence and pseudo‐absence data were set to be equal.

### 2.2 Variable Collection and Data Processing

Considering the biological traits and ecological principles of *Hyphantria cunea* (Ye et al. 2024), we address three primary influences: climate, ecology, and human factors.

#### 2.2.1 Variables of Climate and Vegetation Distribution

The research employs the climate dataset from version 2.1 of the WorldClim database (Fick and Hijmans 2017). The dataset encapsulates the monthly average climate conditions from 1960 to 2021, serving as a representative of recent historical climate conditions with a 2.5‐min spatial resolution. The study uses historical monthly average climate data from 2011 to 2020. The vegetation distribution variable includes the latest global remote sensing product, the Normalized Difference Vegetation Index (NDVI), released by Peking University, which is crucial for assessing the impacts and feedback of climate change on a global scale (Li, Cao, et al. 2023).

#### 2.2.2 Human Footprint and Nighttime Light Intensity Variables

The intensification of human activities is affecting ecological processes, leading to notable changes in species distribution and habitats (Ellis and Ramankutty 2008; Pecl et al. 2017). Consequently, global records from the terrestrial human footprint dataset recently issued by China Agricultural University were adopted (Mu et al. 2022). Nighttime light intensity data, considered a proxy for human activity, can quantitatively characterize economic activities. The global nighttime light dataset recently released by Iowa State University (Li et al. 2020) was chosen, with calibration provided by China Agricultural University. The time range is consistent with the climate variable data.

#### 2.2.3 Future Data for Natural and Anthropogenic Factor Variables

In exploring future climate predictions, this study focuses on two periods from 2021 to 2060: 2021–2040 and 2041–2060. The BCC‐CSM2‐MR (Wu et al. 2019) global circulation model was utilized for evaluation in these phases. For enhanced simulation of climate models and forecasting the diverse possibilities of future climate change, four SSPs were utilized (O'Neill et al. 2016): SSP126, 245, 370, and 585. All predicted data retain the same resolution as the existing climate data in the WorldClim database.

Accurately predicting future vegetation distribution, human footprint, and nighttime light intensity is a critical component of this method, essential for analyzing the future habitat suitability of *H. cunea* . Time series models and random forest algorithms are widely used as methods for predicting variables (Guo et al. 2024; Tziokas et al. 2023; Janković et al. 2021). However, these two algorithms encounter issues of vanishing gradients and non‐stationarity in predicting future variable trends. Consequently, this research uses a combination of time series models and random forest algorithms to acquire future data.

Based on the databases described in sections Method, long‐term historical data sets from 2011 to 2020 were obtained to estimate future distributions of vegetation, human footprints, and nighttime light intensities. Time convolutional networks (TCN) and random forest algorithms were used to complete the forecasting tasks. 80% of the sample data was used for model training, 10% for testing to evaluate the model, and 10% of the total data volume was set aside as a validation set. Through several iterations, the models used were applied to make projections up to the year 2060, eventually yielding future data for three variables.

Climate variables are presented on an annual monthly basis, and processed to obtain annual data to enhance the accuracy of model predictions. To standardize the resolution of the six influencing factors for the four variables, they were resampled and projected, then stored as .asc type files. The processing of future data first involves conversion to .csv file format, followed by prediction using the method described in section Method, and finally, it should be processed into raster file format for storage. Using ArcGIS software, this study processed and stored each of the indicators mentioned above; their identifiers in the model were listed (detail in Table S3).

#### 2.2.4 Correlation Analysis of Variables

**FIGURE 3:**
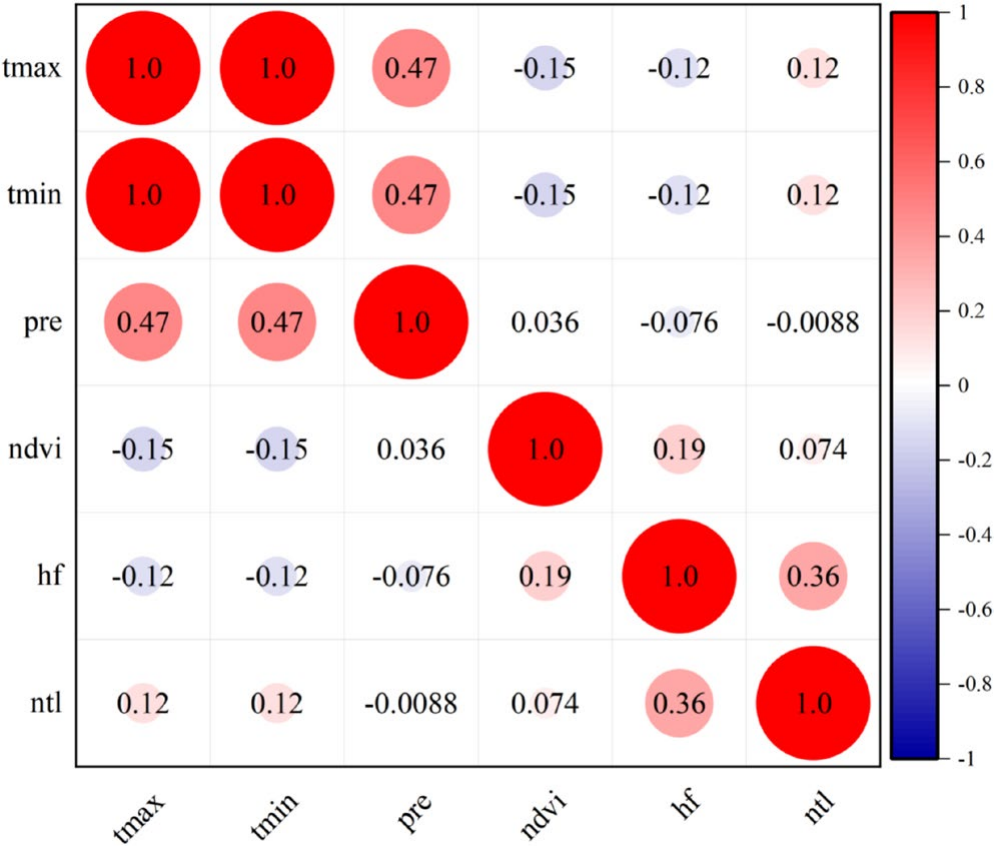
Spearman correlation coefficient matrix for testing the relationships among variables.

To ensure a high degree of independence among the selected environmental variables and to avoid information redundancy caused by strong correlations between similar or closely related variables, this study employed the Spearman correlation analysis to assess the independence of all research variables. This method is well‐suited for evaluating monotonic relationships and is particularly effective for data that are not normally distributed or exhibit nonlinear patterns. By constructing a correlation coefficient matrix, the relationships among the environmental variables were systematically revealed and visually presented, as shown in Figure 3.

As shown in the figure, both positive and negative correlations exist among the variables involved in the study. However, the correlation coefficients between variables are all within the range of ±0.5, indicating that there are no strongly correlated variable pairs. This result suggests that the selected variables exhibit a high degree of independence, providing relatively distinct and complementary information for subsequent modeling. Such independence is beneficial for enhancing the stability and predictive performance of the model.

### 2.3 Model Design

To explore the effectiveness of other regression models compared with the Maxent model, we leveraged the strengths of different algorithms to reduce the bias of a single model and enhance the stability and reliability of predictions. Using the Biomod2 niche modeling framework, we ran 10 different algorithms within a single framework, including Maxent, Random forest (RF), Generalized linear models (GLM), Artificial neural networks (ANN), Classification and regression trees (CRT), Flexible discriminant analysis (FDA), Generalized additive models (GAM), Gradient boosting machines (GBM), Multivariate adaptive regression splines (MARS), and Surface range envelope (SRE). The results were then comprehensively evaluated.

To ensure the model's accuracy and reliability, background point data are randomly assigned to 25% as a test set and 75% as a training set. Furthermore, the model is iterated 10 times using the same method, with the output format set to “Logistic” and the maximum number of iterations fixed at 500. To avoid overfitting, this study incorporates random elements into the model application and implements a cross‐validation strategy.

Real skill statistics (True skill statistics, TSS) have been widely used in the evaluation of models. The AUC (Area Under the Curve) is a metric used to evaluate rank correlation, with its values ranging from 0 to 1. When values approach 1, they indicate a stronger correlation between the chosen variables and the species' geographic distribution, suggesting greater predictive accuracy of the model (Teng et al. 2023). Specifically, AUC values between 0.5 and 0.7 indicate low predictive precision of the model, values between 0.7 and 0.9 indicate moderate predictive precision, and values over 0.9 indicate high predictive precision. If the AUC value is below 0.5, it means the model is too random and the prediction is poor (Jim'enez‐Valverde 2012; Swets 1988).

In incorporating American white moth distribution data and six influencing factors into the model analysis, this study follows these specific computational steps: 75% of observed distribution points are selected as the dataset for building the model, while the remaining 25% is reserved for model validation. This procedure is repeated 10 times to guarantee the results' stability and reliability. Ultimately, the area under the receiver operating characteristic (ROC) curve (AUC) is used as an evaluation metric to quantify the model's predictive accuracy.

## 3 Results

### 3.1 Assessment of the Model Prediction Accuracy

The AUC and TSS values predicted by individual models are shown in Table 1. The results indicate that among the individual models predicting the suitable habitats of the gypsy moth in the United States, the Maxent model performed the best, while the Surface Range Envelope (SRE) model had the poorest prediction performance. To improve the prediction accuracy of the ensemble model, among the 1000 training results, 842 results with AUC ≥ 0.9 and TSS ≥ 0.85 were retained. Using their AUC values as weights, an ensemble model was constructed using the weighted average method. The final prediction results of the ensemble model (based on GLM and Maxent) yielded an AUC of 0.931 and a TSS of 0.877, indicating that the evaluation metrics of the ensemble model were slightly lower compared to the individual Maxent model.

**TABLE 1:** Average evaluation index of single models.

	RF	GLM	ANN	CRT	FDA	GAM	GBM	MARS	SRE	Maxent
AUC	0.894	0.904	0.884	0.839	0.896	0.898	0.877	0.852	0.851	0.939
TSS	0.866	0.876	0.821	0.848	0.878	0.879	0.857	0.859	0.703	0.884

### 3.2 Current Global Distribution of *Hyphantria cunea*

**FIGURE 4:**
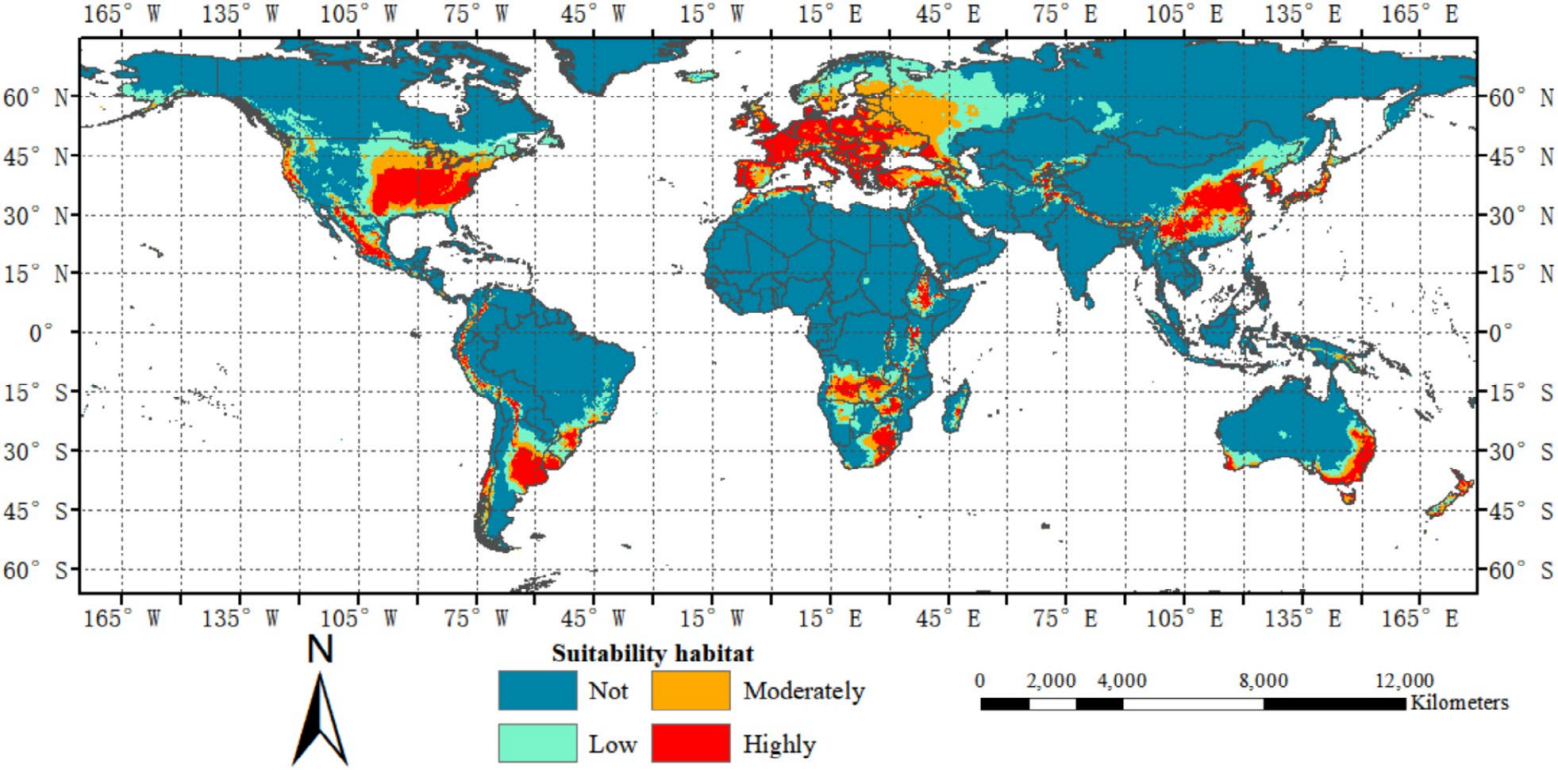
Predictive analysis of the potential global distribution of the fall webworm ( *Hyphantria cunea* ) under current conditions.

This research incorporated observed distribution points along with indices of natural and human factors into the Maxent model, resulting in a depiction of the current potential global distribution of *Hyphantria cunea* , as illustrated in Figure 4.

The suitability habitat of *Hyphantria cunea* has been demonstrated worldwide. Europe offers suitable habitats for this pest in most of its regions. Highly suitable habitat in Asia encompasses China, South Korea, North Korea, Japan, the north of India and Pakistan, Myanmar, Bhutan, Nepal, Afghanistan, Uzbekistan, Tajikistan, and the fringes of Kazakhstan. The authors found highly suitable habitat for the pest solely in central and southern Africa. In Oceania, southeastern and southwestern coastal areas of Australia and parts of New Zealand show highly suitable habitat. Significant suitability for *H. cunea* is exhibited in the United States, Mexico, and along the southeastern border of Canada in North America. The distribution pattern in South America is primarily focused on the western coastal countries, Argentina, Uruguay, Bolivia, and the southern borders of Brazil, showing a bilateral distribution.

### 3.3 The Impact of Variable Indicator Factors on the Current Distribution of *Hyphantria cunea*

All occurrence records in the test samples are located at coordinate points, and the geographic distribution (Figure 2) is highly consistent with the visual modeling predictions (Figure 3). Therefore, the predicted current distribution of *Hyphantria cunea* shows a significant positive correlation with the test data. According to Figure 4, *H. cunea* is suitable between 52° 23 min south latitude and 60° 28 min north latitude.

**FIGURE 5:**
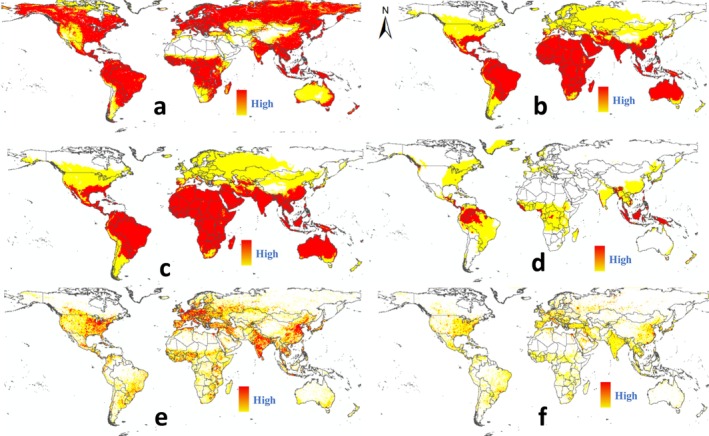
Historical distribution of variable index factors: (a) NDVI; (b) Average maximum temperature; (c) Average minimum temperature; (d) Average precipitation; (e) Human footprint; (f) Nighttime light intensity.

Based on the predicted distribution, the *Hyphantria cunea* demonstrates high suitability across most global regions. Comparing the potential global distribution of *H. cunea* (Figure 4) with historical occurrences of variable indicators (Figure 5) reveals overall correlations. The distribution of *H. cunea* generally depends on the presence of host plants, such as crops and fruit trees (Edosa et al. 2018). The NDVI in Figure 5a, indicating global vegetation cover, correlates with the distribution of host plants for *H. cunea* . Unsuitability in northern Africa, the Middle East, Mongolia, and northwestern China might relate to lower levels of vegetation cover. Observations from Figures 4 and 5b–d show that climatic factors also affect the suitability distribution of *H. cunea* , though the overall correlation is low. In contrast, human factors demonstrate a strong correlation with the suitability distribution of *H. cunea* , particularly where the distribution of human footprint (Figure 5e) matches well with *H. cunea* 's current global geographical areas. Therefore, human factors may be the main reason affecting the global suitability distribution of *H. cunea* .

### 3.4 Analysis of Variable Indicator Factors

**FIGURE 6:**
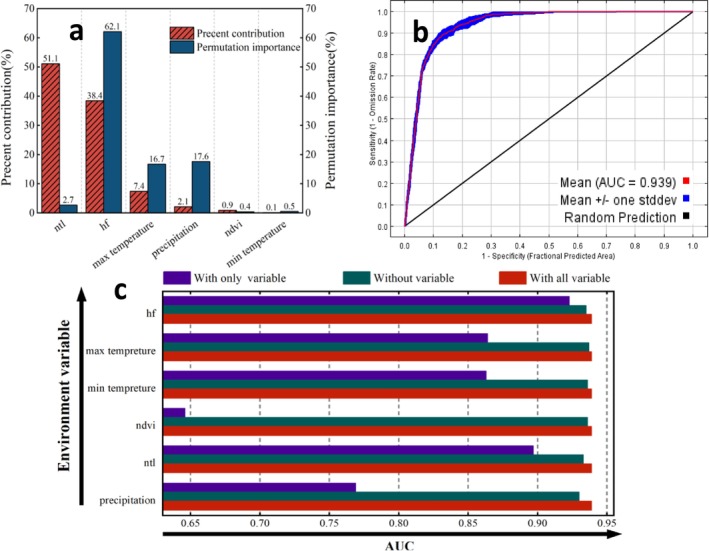
Analysis of influence factors and model indicators. (a) When predicting the distribution of *Hyphantria cunea* using the Maxent model, the contribution of variable indicators to the model prediction is expressed as a percentage, and the percentage importance of these factors is also assessed; (b) results of Jackknife validation analysis for predicting the potential distribution of *H. cunea* using the Maxent model; (c) validation of the ROC curve for Maxent's predictions of point distributions of *H. cunea* .

In order to delve deeper into the relationship between the potential distribution of *H. cunea* and every indicator variable, quantitative evaluations were performed using the percentage contribution and permutation importance metrics, as shown in Figure 6a.

As shown in Figure 6a, the intensity of night lights and human footprints have the best fit effect on the model, indicating that these two variable factors have a high contribution percentage in the model, reaching 51.1% and 38.4% respectively. Human factors play a more significant role in the model's predictive capabilities, particularly as the importance of natural factors comprises only 35.2%. Moreover, human factors have the greatest effect on the predictions, notably the human footprint variable, which constitutes 62.1% of the importance.

The results of the analysis of all variable indicator factors were further validated through a Jackknife test, as shown in Figure 6c. From Figure 6c, it can be seen that all variable factors contribute to the correlation with the model's predictions, particularly human factors, which have the most pronounced impact. Specifically, when only the human footprint (hf) variable factor is included, the model's AUC value is at its highest, indicating that this variable alone contains a significant amount of critical information about the potential distribution of *H. cunea* .

During the development of the distribution model for *H. cunea* , the model's predictive performance was accurately assessed by combining the AUC (Area Under the Curve) values from 10 iterations of the Maxent model (detail Table S1). These AUC values are crucial for gauging the accuracy of the model in predicting species distributions. Figure 6b presents the Receiver Operating Characteristic (ROC) curve used in this study for predicting the distribution of *H. cunea* . From the analysis of this curve, the model's average AUC value of 0.939 demonstrates its high accuracy in forecasting the potential distribution areas of *H. cunea* .

### 3.5 Future Global Distribution Prediction Changes

**FIGURE 7:**
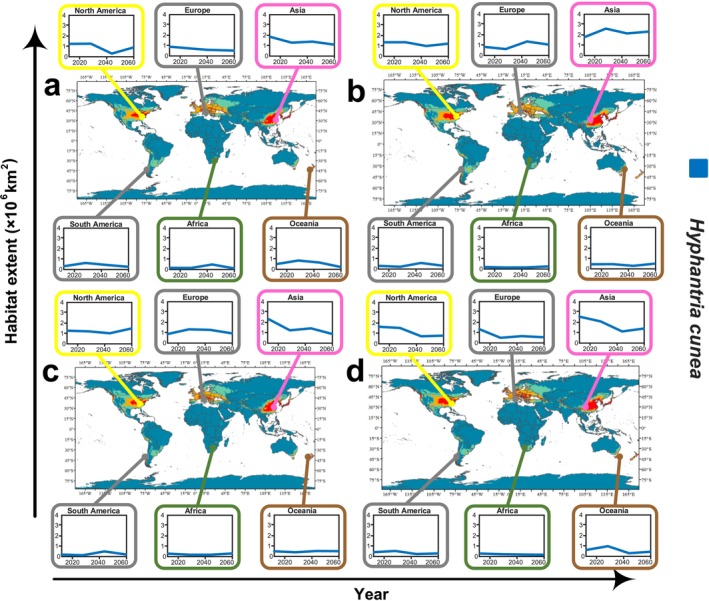
Distribution of *Hyphantria cunea* over the next 40 years and changes in highly suitable habitat across continents (a–d).

In order to further quantify the potential global distribution changes of *H. cunea* , this study utilizes historical data of natural and human factors to forecast distribution scenarios over the next 40 years, including the NDVI, hf, and ntl, as shown in Figure 7.

Through a lateral comparison of predictions from four SSP (126, 245, 370, and 585) between 2021 and 2040 (detail in Table S2), this research observes a trend of initial decline and subsequent increase in the global suitable habitat area for *H. cunea* . Additionally, the proportion of areas unsuitable for survival exhibits no significant large changes in the future, with the expansion and contraction of suitable habitat ranges visually depicted in (detail in Figure S1). Under the SSP126 and 585 scenarios, North America (including the United States and Mexico) and Europe exhibit a trend of reducing suitable habitat area for the pest from 2041 to 2060; however, this trend is not observed to continue in the SSP245 and 370 scenarios (detail in Figure S2).

By conducting longitudinal analyses of various SSP scenarios, the future global suitable habitats for this pest are anticipated to experience more extensive expansion and narrower contraction (detail in Figure S3). During the periods 2021–2040 and 2041–2060, the climatic suitability for *H. cunea* in Europe will show a downward trend (detail in Figure S3). In comparison, in Asia, particularly in China, a notable increase in suitability has been observed, especially pronounced under the SSP245 and 370 scenarios (detail in Figure S3). In the SSP126 and 585 scenario analyses, the geographical distribution expansion of this pest is primarily concentrated in the Northern Hemisphere, involving North America, Europe, and Asia, with large new habitats expected to form in Canada, Belarus, the United Kingdom, and North Korea (detail in Figure S3).

However, in all scenarios, the impact of *H. cunea* on the Southern Hemisphere is equally significant, with predictions showing that extensive moderately to highly suitable habitats will appear in South American countries such as Chile, Argentina, southern Brazil, Bolivia, Peru, and Uruguay. While New Zealand's suitability does not exhibit a marked decline, Australia is expected to demonstrate increased suitability in the future, with the Republic of South Africa encountering similar circumstances. This analysis highlights the dynamic changes in the suitability of habitats for this pest in different regions globally under the dual changes of natural and anthropogenic factors in the future, further emphasizing the importance of considering these prediction models when formulating targeted management and adaptation strategies.

Considering the combined impacts of natural and human factors along with SSP scenarios, the original distribution areas of *H. cunea* in North America are projected to undergo a decrease in habitat suitability. In Europe, although signs of pest spread are observed, the potential expansion of highly suitable habitat areas is expected to be limited, primarily targeting the Nordic regions. For Oceania, Africa, and South America, the potential suitable habitat areas in these regions are significantly less compared to Asia, with New Zealand being the only country showing a larger suitable area. In Asia, most countries including North Korea, Japan, South Korea, and China show high adaptability to *H. cunea* , but preliminary surveys in North Korea have not shown clear signs of pest invasion. Especially in China, the Asian country most significantly impacted by *H. cunea* , significant habitat changes are anticipated in the future due to shifts in natural and human factors and economic model transitions, showing a clear trend of expansion.

## 4 Discussion

Biological invasions are one of the major threats to global biodiversity and ecosystem functions. Several species have demonstrated aggressive behaviors, and it is anticipated that their populations will not diminish in the future (Divíšek et al. 2018). The suitable habitat area for *H. cunea* is expected to expand globally in the future. However, the expansion trend varies between the Northern and Southern Hemispheres. The Northern Hemisphere may continue to serve as the primary habitat for *H. cunea* . Conversely, the same trend of expansion is not evident in South America and Africa in the Southern Hemisphere. This phenomenon highlights the necessity of enhancing surveillance of *H. cunea* invasion in countries of the Northern Hemisphere. Moreover, there is a need for continued vigilance in Oceania, including Australia and New Zealand.

Previous studies have indicated that climate change has shown impacts on the suitability for *H. cunea* at various national scales (Kean and Kumarasinghe 2007; Tang et al. 2021; Wen et al. 2024), yet this is rarely discussed on a global level. Compared to the global predictions by Ge et al. (2019), this paper's findings suggest that human factors are the primary drivers of changes in habitat suitability for *H. cunea* , aligning with the views of Ye et al. (2024). Ge et al. (2019), employing the CLIMEX model, only included climatic factors to forecast the potential global distribution of *H. cunea* , and did not thoroughly analyze NDVI or human factors. The CLIMEX model utilizes biological parameters to depict the responses of species to climatic variables over varying time scales. However, the biological characteristics of species differ across various environmental regions, and climate factors are just one of the elements affecting species distribution, which can reduce the accuracy of the models. Although Ye et al. analyzed the impact of human factors on the spread of *H. cunea* , their simulation focused narrowly on human influences and insufficiently considered the study area (limited to China).

This paper specifically includes not only climatic factors but also the geographic distribution of NDVI and human influences. Research indicates that the model's predictions correspond closely with the actual known distribution areas of *H. cunea* , demonstrating the model's high accuracy in forecasting its distribution. In Europe, the majority of regions are recognized as potential suitable habitats, aligning with the recorded distributions. Especially in Asia, including China, Japan, and Korea, the invasion of *H. cunea* is notably severe, which is supported by the model's predicted potential distributions. The authors consider this distribution phenomenon to be due to dense human footprints. Additionally, the model also predicts potential distributions of *H. cunea* in parts of Africa, South America, Australia, Korea, and Central Asia, areas currently not recorded with invasions, suggesting that its potential distribution might exceed the current observational range. Regions lacking NDVI, such as North Africa, the Middle East, and the Tibetan Plateau in China, are unsuitable for the survival of *H. cunea* with study results adhering to the pest's dissemination requirements (necessitating host plants). Earlier research asserted that climate change would diminish the suitable habitat area for *H. cunea* in the Southern Hemisphere (Ge et al. 2019), especially in Oceania, including Australia. This paper's results are starkly different, even contrary, from previous studies; similarly, the authors have also shown that New Zealand is one of the highly suitable regions for *H. cunea* , a point not indicated in earlier research. In the long term, the SSP126 and 585 scenarios forecast a reduction in the suitable living area for *H. cunea* by 25.2% and 33.2%, respectively, whereas the SSP245 and 370 scenarios predict increases of 13.9% and 5.7%, respectively (detail in Table S2). Although nighttime light intensity reflects human activity patterns well, it has not yet shown sufficient significance compared to human footprints.

Climate change is expected to further exacerbate the severity of invasive pests, as temperature variations could facilitate their establishment and spread (Pautasso et al. 2010; Jepsen et al. 2011; Seidl et al. 2018). At the macro level, climate is a key factor, mainly related to temperature and precipitation (Shi et al. 2010; Czarniecka‐Wiera et al. 2020); however, social factors significantly impact the distribution of invasive species (Zhou et al. 2020), especially as human activities play a crucial role in the intentional or unintentional introduction of these species (Xiang et al. 2023; Sun et al. 2023). Frequent human activities and rising economic development facilitate the easy introduction of *H. cunea* . The distribution of hosts is one of the determinants of pest suitability, and the long‐distance transmission of pests is closely related to human factors, both of which should be discussed. This assertion has also been confirmed in this study. Thus, objectively, human factors are the primary drivers influencing the distribution of *H. cunea* , more so than natural factors.

## 5 Conclusion

This study utilizes the Maxent model, incorporating human and natural factors to predict the current and future potential global distribution of *H. cunea* , for comparison with climate change. Results indicate that under the influence of climate change, human factors have significantly altered the potential global distribution of *H. cunea* . In contrast to the potential distribution driven by climate change, this paper suggests that the suitable habitat area for *H. cunea* in Oceania, Southern Hemisphere, is expected to increase. It is important to note that the distribution of NDVI is a crucial factor affecting the suitability of *H. cunea* ; areas lacking host plant distribution are unsuitable for its survival. Additionally, the authors consider that the long‐distance dispersal of pests is closely linked to human factors. In the long term, although nighttime light intensity well reflects human activity patterns, it has not yet shown enough significance compared to human footprints. This study emphasizes the importance of incorporating human factors into the prediction of invasive pest distributions under climate change. Using *H. cunea* as an example, this paper's results provide a comprehensive perspective on how human factors interact with climate change to affect the global suitability distribution of *H. cunea* . Given the potential expansion of *H. cunea* 's distribution, countries in highly suitable areas should establish and strengthen quarantine programs and control strategies to prevent further introduction and spread of *H. cunea* , thereby protecting biodiversity and preventing further ecosystem function degradation.

## Author Contributions

**Haochang Hu:** methodology (lead), resources (lead), software (lead), validation (lead), visualization (lead), writing – original draft (lead), writing – review and editing (equal). **Hongwei Zhou:** conceptualization (lead), funding acquisition (lead), investigation (lead), project administration (lead), writing – review and editing (lead). **Yuxi Li:** data curation (equal), formal analysis (equal), resources (equal), software (equal), validation (equal). **Yongzheng Li:** data curation (equal), formal analysis (equal), methodology (equal), resources (equal), visualization (equal). **Yunbo Yan:** investigation (equal), validation (equal). **Jun Yang:** data curation (equal), supervision (equal). **Jun Chen:** validation (equal). **Yumo Chen:** validation (equal). **Di Cui:** visualization (equal).

## Conflicts of Interest

The authors declare no conflicts of interest.

## Supporting information


Data S1.

Data S2.

## Data Availability

The data used in this study are included in the Supporting Information.
