# Supplementary material for: The Involvement of Human Factors Brings New Findings for Predicting Global Suitability Habitat for Hyphantria cunea (Lepidoptera: Arctiidae)
Source: Ecol Evol. 2025 May 26;15(5):e71421. doi: 10.1002/ece3.71421 (PMC12104870; doi:10.1002/ece3.71421)
Supplement: Supplementary file 1 — Data S1. [file ECE3-15-e71421-s002.docx]

**Supplementary Information**

**Supplementary Information**

**Tab. S1: Ten statistical analysis results of the predictive performance for *Hyphantria cunea* distribution within Maxent, assessed using AUC.**

| Species | AUC | Standard Deviation |
| --- | --- | --- |
| *Hyphantria cunea* | 0.9412 | 0.0074 |
|  | 0.9394 | 0.0084 |
|  | 0.9422 | 0.0064 |
|  | 0.9346 | 0.0094 |
|  | 0.9346 | 0.0078 |
|  | 0.9403 | 0.0063 |
|  | 0.9344 | 0.0092 |
|  | 0.9436 | 0.0061 |
|  | 0.9334 | 0.0107 |
|  | 0.9415 | 0.0076 |

**Tab. S2: Area of global suitability changes for *Hyphantria cunea* under different climate scenarios.**

| Suitability | Unsuitable(×10^8^km^2^) | |  | Lowly suitable(×10^7^km^2^) | |  | Moderately  suitable(×10^7^km^2^) | | | Highly suitable(×10^6^km^2^) | | |  |
| --- | --- | --- | --- | --- | --- | --- | --- | --- | --- | --- | --- | --- | --- |
| Year | 21-40s | 41-60s |  | 21-40s | 41-60s |  | 21-40s | 41-60s |  | | 21-40s | 41-60s | |
| SSP126 | 1.82 | 1.86 |  | 1.82 | 1.62 |  | 1.12 | 1.09 |  | | 4.96 | 3.20 | |
| SSP245 | 1.83 | 1.83 |  | 1.75 | 1.74 |  | 1.06 | 0.97 |  | | 4.61 | 5.91 | |
| SSP370 | 1.84 | 1.83 |  | 1.76 | 1.81 |  | 1.01 | 1.01 |  | | 4.72 | 5.13 | |
| SSP585 | 1.83 | 1.85 |  | 1.72 | 1.69 |  | 0.96 | 1.08 |  | | 6.47 | 3.34 | |

The suitability is classified into four levels using the Natural Breaks classification method, with the threshold ranges as follows: Unsuitable (0.00–0.20), Lowly suitable (0.21–0.50), Moderately suitable (0.51–0.80), and Highly suitable (0.81–1.00).

**Tab S3: All types of variables and indicators used in the study.**

| Type of variable | Name of index | Code name in the model |
| --- | --- | --- |
| Natural factor | average annual maximum temperature | max temperal |
|  | average annual minimum temperature | min temperal |
|  | mean annual precipitation | precipitation |
|  | NDVI | ndvi |
| Human factor | human footprint | hf |
|  | nighttime light intensity | ntl |

**Fig. S1: Horizontal comparison of predicted results for *Hyphantria cunea* under four scenario models from 2021 to 2040. (a) SSP126; (b) SSP245; (c) SSP370; (d) SSP585.**

**Fig. S2: Horizontal comparison of predicted results for *Hyphantria cunea* under four scenario models from 2041 to 2060. (a) SSP126; (b) SSP245; (c) SSP370; (d) SSP585.**

**Fig. S3: Prediction results of the suitability of *Hyphantria cunea* habitat under different shared socioeconomic pathways in future climate scenarios. (a) SSP 126 and 245 scenario prediction result; (b) SSP 370 and 585 scenario prediction results.**

**Data**

- Global Biodiversity Information Facility(http://www.gbif.org/)
- Barcode of Life Data Systems(http://www.boldsystems.org/)
- Centre for Agriculture and Bioscience International(https://www.cabi.org/isc)
- WorldClim(https://www.worldclim.org)
- Normalized Vegetation Index^1^
- Human Footprint^2^
- global nighttime light^3^
- Beijing Climate Center Climate System Model^4^
- CMIP6^5^

**References**

1. Li, M. et al. Spatiotemporally consistent global dataset of the GIMMS Normalized Difference Vegetation Index (PKU GIMMS NDVI) from 1982 to 2022, Earth Syst. Sci. Data, 15, 4181–4203 (2023).
2. Mu, H. et al. A global record of annual terrestrial Human Footprint dataset from 2000 to 2018. Sci Data 9, 176 (2022).
3. Li, X. et al. A harmonized global nighttime light dataset 1992–2018. Sci Data 7, 168 (2020).
4. Wu T et al., The Beijing Climate Center Climate System Model (BCC-CSM): the main progress from CMIP5 to CMIP6. Geosci. Model Dev, 12:1573–1600 (2019).
5. O’Neill B C et al., The scenario model intercomparison project (ScenarioMIP) for CMIP6. Geosci. Model Dev, 9:3461–3482 (2016).

**Supinfo1**
